# Impact of Autogenous Demineralized Dentin Matrix on Mandibular Second Molar after Third Molar Extraction: Retrospective Study

**DOI:** 10.3390/jfb14010004

**Published:** 2022-12-20

**Authors:** Yesel Kim, Jeong-Kui Ku, In-Woong Um, Hyun Seok, Dae Ho Leem

**Affiliations:** 1 Department of Dental Hygiene, Jeonju Kijeon College, Jeonju 54989, Republic of Korea; 2 Department of Oral and Maxillofacial Surgery, School of Dentistry and Institute of Oral Bioscience, Research Institute of Clinical Medicine of Jeonbuk National University, Biomedical Research Institute of Jeonbuk National University Hospital, Jeonbuk National University, Jeonju 54907, Republic of Korea; 3 R&D Institute, Korea Tooth Bank, Seoul 06101, Republic of Korea

**Keywords:** bone graft substitutes, demineralized dentin matrix, third molar, tooth extraction

## Abstract

The purpose of this retrospective study was to evaluate bone healing after autogenous demineralized dentin matrix (DDM) grafts, focusing on the distal root of the mandibular second molar after the extraction of the third. We included retrospective data from 20 patients who had undergone molar extractions (15 male, 41.9 ± 12.0 years) between January 2020 and September 2022 and had DDM grafts implanted on the extraction socket, immediately (“immediate graft”) or 6 weeks (“delayed graft”) after the first surgery without primary closure. Patients who underwent grafting on only one side were used as the control group (*n* = 4). Bone defects at the mandibular second molar were measured preoperatively and 4 months after the graft surgery using cone-beam computed tomography (CBCT). Improvement of bone defect (i.e., the change in the bony defect pre- vs. postoperatively) was compared between the control and graft groups using the Wilcoxon Signed Rank test, and the difference between immediate and delayed grafts was analyzed with the Mann-Whitney U test. Complications such as infections or graft failure did not occur. Although pre-operative defects were smaller in the control than in the graft group (2.98 ± 1.77 and 10.02 ± 3.22 mm, *p* = 0.001), post-operative defects were similar in both (2.12 ± 0.59 and 2.29 ± 1.67 mm, respectively). The improvement ratio was not statistically significant in the control group (22.68 ± 15.36%) but a difference was observed in the graft group (76.70 ± 15.36%, *p* = 0.001). The amount of improvement of bone defect was not affected by graft timing or patient sex. In conclusion, DDM can improve bone defect at the distal aspect of the mandibular second molar after third molar extraction.

## 1. Introduction

Third molar extraction is a common oral surgery procedure in daily dentistry practice. As individuals show large anatomic variations in this tooth, its extraction occasionally affects the periodontal status of the adjacent second molar. Extensive studies have shown periodontal complications at the mandibular second molar (M2) after extraction of the third (M3) [1], and a microbiology evaluation found that the microflora in the operculum of an M3 that was not fully erupted due to a horizontal impaction was similar to that detected during chronic periodontitis [2].

Kugelberg found that about 32% of M3 extraction patients eventually remained with an alveolar bony defect of more than 4 mm at the distal aspect of the second molar [3]. Low et al. reported in their systematic review and meta-analysis for M2 healing after M3 extraction that residual pockets still remained at 5 mm in patients > 25 years of age with an initial pocket depth (PD) > 7 mm [4]. Bone grafting has been recommended to extract a third molar that has a large contact area with the second molar in patients over the age of 25 to prevent periodontitis at the second molar [5,6]. However, inconsistent effects of bone graft surgeries at M2 using various biomaterials have been reported [7]. Some studies found that the use of a collagen sponge facilitated postoperative wound healing and prevented periodontal defects at M2 [8]. However, they assessed bone healing at M2 with 3D rather than 2D measurement techniques, which analyzed bone density, and obtained different results from actual bone healing on computed tomography (CT) [9]. In 2021, Ku and Jeong reported on the use of CT to measure bone healing at the distal root of M2 after M3 extraction and found that demineralized bone matrix (DBM) enriched with recombinant human bone morphogenetic protein-2 (rhBMP-2) led to significant improvements compared to a collagen plug [10]. As findings remain controversial due to the cost-effectiveness of using rhBMP-2, an efficient bone graft material for M3 extractions still needs to be developed.

Despite its resorption tendency and propensity for donor site morbidity, autogenous bone is the gold standard for bone graft surgeries for its osteoconduction, osteoinduction, and osteogenesis properties [11]. Tooth-derived bone matrix, DDM, has recently been shown to possess osteoinductivity and an efficacy comparable to that of autogenous bone [12,13]. Some research on DDM demonstrated similar radiographic and histological outcomes compared with autogenous bone and bovine-derived xenograft [14], and no inferior volumetric effect compared with deproteinized bovine bone with collagen [15]. In 2020, Sánchez-Labrador et al. conducted a clinical split-mouth trial and reported that the use of mineralized dentin matrix (MDM) resulted in a statistically significant reduction in pocket depth at both 3 and 6 months post-surgery, but found no difference in bone healing [16]. DDM has also been shown to induce new bone formation to a larger degree than mineralized dentin [17]. The authors hypothesized that DDM could improve the bone defect of M2 without any complications. The aim of this study was to further evaluate the improvement of bone defect induced by DDM, using 3D measurement techniques with a focus on the distal root of M2 after M3 extraction.

## 2. Materials and Methods

All procedures described in this retrospective study were in accordance with the Helsinki Declaration as revised in 2008. The study was approved by the Jeonbuk National University Hospital Institutional Review Board (IRB No. 2022-09-032). Informed consent was obtained from all patients.

Patients who visited the Department of Oral and Maxillofacial Surgery at Jeonbuk National University from January 2020 to September 2022 were included in this study. They were selected through a review of medical records and included on the basis of the following inclusion criteria: (1) age > 25 years; (2) no history of systematic disease or oral/maxillofacial syndrome, injury, or surgery; (3) no history of smoking or drug use; (4) pre- and post-operative CT scans, the latter obtained at least 3 months after the extraction; (5) pre-operative bone defects on CT > 4 mm, based on the cementoenamel junction (CEJ) of the mandibular second molar; (6) a bone graft with autogenous DDM implanted within 6 weeks after the extraction. The exclusion criteria were as follows: patients with active periodontitis or root caries of M2.

Acquisition parameters of CBCT (Alphard 3030, Asahi Roentgen Ind., Kyoto, Japan) were as follows: 80 kV, 8 mA, 17 s exposure time, 154 × 154 mm field of view, and 512 basis projections. Each scan was reconstructed and saved in a standard DICOM file with a voxel size of 0.1 mm. All images were transferred to a picture-archiving and communication system viewer (INFINITT PACS, Infinitt Healthcare, Seoul, Republic of Korea). The images were reformatted CT data to a panoramic view based on the mandibular arch (middle of mandibular teeth and inferior mandibular canal) with an axial cut and selected single image cut, which included the middle of the extracted third molar [18]. On the cross-sectional view of CBCT, a vertical reference line was the distal root surface of the second molar. The bony defect was measured based on the exposure of the second molar from the CEJ of M2 [10]. The specific threshold value (370–3071 Hounsfield units for bone demonstration) was reconstructed for measurement [9]. The measurement was performed by one examiner (Y.Kim) twice at 2 weeks intervals, and averaged. The inter-examiner coefficient was 0.992 between the measurements. (*p* < 0.001)

### 2.1. Study Design

The autogenous DDM (Auto-DDM) used in this study was manufactured at an independent institute (AutoBT^®^, Korea Tooth Bank, Seoul, Republic of Korea) using each patient’s extracted tooth. Briefly, the extracted human teeth were cleaned by removing the soft tissues with 70% ethyl alcohol. The root portion was collected and crushed. The crushed particles were treated in distilled water and hydrogen dioxide solution. The cleaned particles were dehydrated with ethyl alcohol and ethyl ether solution. The particles were then demineralized for 30 min in 0.6 N HCl. The demineralized particles were lyophilized, packed, and sterilized with ethylene oxide gas [19]. Since this process as well as delivery to our department required a time of about 2 weeks, two different protocols were employed: “immediate” grafting and “delayed” grafting, depending on whether pre-manufactured Auto-DDM from the other tooth could be prepared before M3 extraction. Patients who received a graft on only one side and a collagen plug on the other were used as a control group. All procedures were performed by the same surgeon.

#### 2.1.1. Immediate Graft

**Figure 1:**
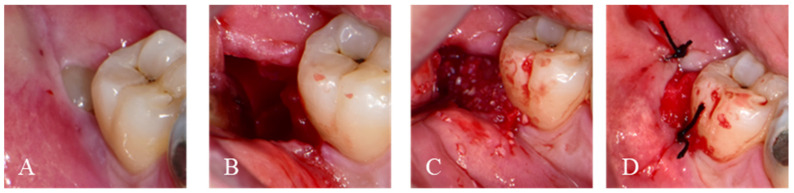
Infraoperative images for immediate graft of Auto-DDM with M3 extraction. (**A**). Horizontally impacted third molar. (**B**). After extraction, alveolar defect on the adjacent second molar (**C**). Auto-DDM was grafted on the distal root surface of M2. (**D**). The graft was covered with a collagen plug without primary closure.

Patients with other teeth to be extracted as well, such as the maxillary third molar, underwent grafting immediately after the third mandibular extraction using pre-manufactured Auto-DDM. Our surgery protocol followed that of a previous study [10]. Briefly, after routine draping and local anesthesia (Lidocaine HCl, Huons, Seongnam-Si, Republic of Korea), an incision was made following the operculectomy protocol, and the third molar was extracted using a high-speed 2.0-mm round burr. After complete curettage of the remnant tissue, Auto-DDM was grafted onto the exposed distal root surface of the second molar and covered with a collagen plug (Figure 1). Two sutures were placed on the mesiodistal margins of the incision with 4-0 Vicryl (Ethicon, Johnson & Johnson International, New Jersey, USA) without a water-tight suture.

#### 2.1.2. Delayed Graft

**Figure 2:**
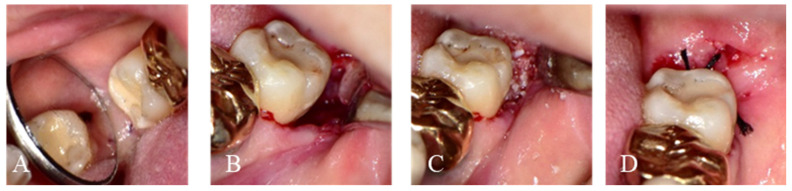
Intraoperative images for delayed graft of Auto-DDM after 6 weeks of M3 extraction. (**A**). Incomplete healing of alveolar defect on the adjacent second molar. (**B**). Flap elevation and curettage of granulation tissue. (**C**). Auto-DDM was grafted on the distal root surface of M2. (**D**). The graft was covered with a collagen plug without primary closure.

If there was no other tooth to be extracted, the third molar was removed and sent to the Korea Tooth Bank. The graft surgery was performed 6 weeks later, to allow enough time for the manufacturing process of the Auto-DDM as well as the gingival healing period. The distal root surface of the second molar was exposed through an enveloped incision to perform the curettage on the exposed distal root of M2. Auto-DDM was grafted onto the exposed M2 distal root, and then the graft was covered with a collagen plug as described in the immediate graft protocol. One suture was placed on the mesial margin of the incision with 4-0 Vicryl. (Figure 2)

### 2.2. Post-Operative Surgery Protocol

**Figure 3:**
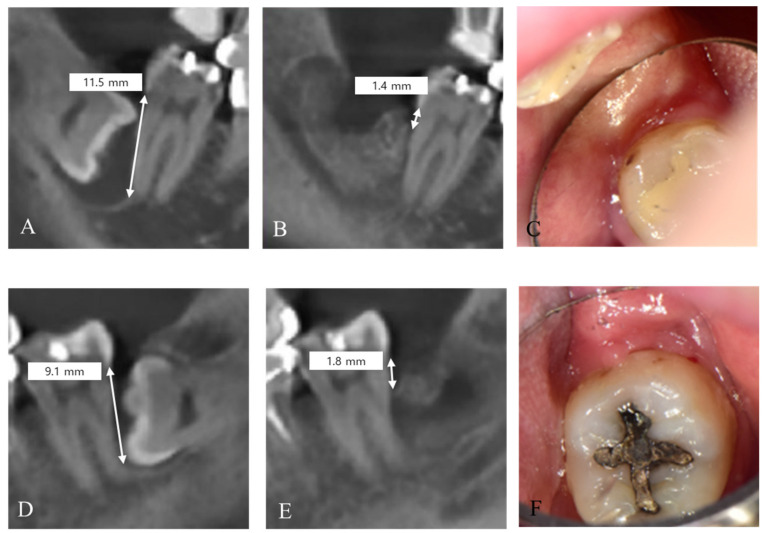
Measurement of bony defect at the distal aspect of the adjacent second molar. (**A**–**C**). Immediate graft. (**D**,**E**). Delayed graft. (**A**,**D**). Pre-operative. (**B**,**E**). Post-operative. (**C**,**F**). Post-operative intraoral images.

Bony defects at the distal aspect of the adjacent second molar were assessed using CBCT and INFINITT PACS. By using CBCT, the bone level was measured from the CEJ of the second molar to the most apical point in a cross-sectional slice, once pre-operatively and once 4 months after extraction. (Figure 3)

### 2.3. Statistical Analysis

The Kolmogorov–Smirnov test was used to analyze parametric data, while pre- vs. post-operative changes in bone defects were analyzed using the Wilcoxon Signed Rank test. Improvement (change from pre-operative to post-operative bone defects) and improvement ratio ($\frac{Improvement\;\left(mm\right)}{Pre-operative\;bone\;defect\;\left(mm\right)}\times 100$) was analyzed using the Mann-Whitney U test to assess differences between the control and graft groups as well as between immediate and delayed grafts. Data are expressed as means ± standard deviations and analyzed using 25.0 IBM SPSS Statistics (IBM, Armonk, NY, USA).

## 3. Results

A total of 20 extraction areas (15 from male patients, 41.9 ± 12.0 years) were included in this study based on medical chart reviews. (Table 1)

**Figure 4:**
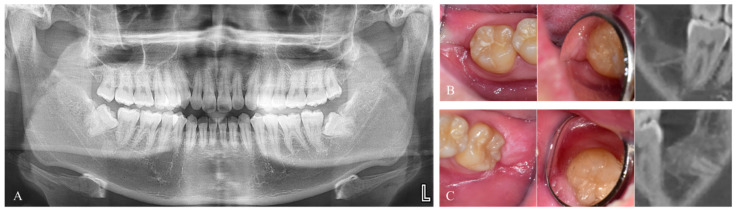
Pre-operative and post-operative images. (**A**). Pre-operative radiograph of a patient who had immediate graft on the left side with Auto-DDM, fabricated from the right mandibular third molar without any graft after the extraction on the right side. (**B**). Post-operative images at four months after the extraction without graft (Control group). (**C**). Post-operative images at three months. after the graft (Graft group).

Four patients (39.3 ± 6.3 years) did not undergo Auto-DDM grafting and were classified as the control group, while the other 16 (42.6 ± 13.3 years) received grafts. (Figure 4)

Complications such as infections or graft failure did not occur. Although the pre-operative defects observed in the control patients (2.98 ± 1.77 mm) were smaller than those in the patients receiving grafts (10.02 ± 3.22), post-operative defects were similar in both groups (2.12 ± 0.59 and 2.29 ± 1.67 mm after 5.5 ± 2.6 and 4.6 ± 2.0 months, respectively, Table 2).

**Figure 5:**
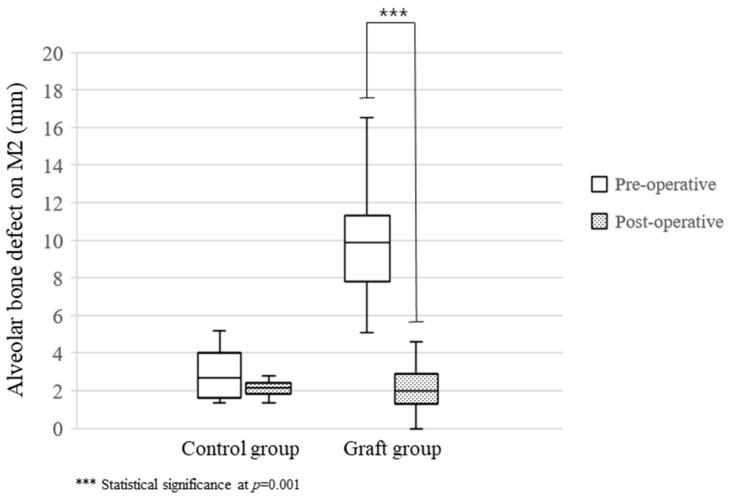
The changes of bone defect on M2 according to the control and Auto-DDM grafted groups. Graft group showed a statistical difference between the pre- and post-operative alveolar bone defect on M2 (*p* = 0.001).

The improvement ratio was significantly higher in the graft than in the control group (77.9 ± 15.3 and 22.7 ± 26.2%, *p* = 0.001, Table 2). The pre- vs. post-operative change in defects was not statistically significant in control patients (*p* = 0.285), but a significant effect was observed in the graft group (*p* = 0.001, Figure 5).

Regarding the graft timing, six surgeries were performed immediately and seven after 6 weeks. Patients in the immediate and delayed graft groups had similar ages, follow-up periods, and pre-operative defects, as well as similar improvement ratios (79.5 ± 12.5% and 74.6 ± 17.8%, respectively, *p* = 0.662, Table 3).

## 4. Discussion

This study investigated whether DDM can improve bone defect at the exposed root surface of the adjacent mandibular second molar after third molar extraction. The DDM grafts showed significant improvement on the distal aspect of M2, even though primary closure was not attempted. The bone healing capacity of DDM was evident in all of our middle-aged patients (average 42.6 years) regardless of graft timing or sex.

While autogenous bone has been shown to be an ideal graft material, only a few studies found that it facilitates bone healing at M2 after M3 extraction. In 2017, a randomized controlled trial demonstrated improved regeneration after particulate autogenous bone grafting following M3 extraction. Patients showed postoperative healing of 5.9- and 5.6-mm defects after 1 week and 12 months, respectively, from pre-operative bone defects (7.3 ± 2.2 mm) [20]. The bone healing ratio (76.7%; 5.6 divided by 7.3 mm) reported for these patients is almost identical to the one we observed in the current study (76.70 ± 15.36%).

A number of studies have provided evidence showing that DDM is comparable to the autogenous bone for bone healing capacity to remodel cortico-cancellous complex. The chemical components of dentin are almost identical to those of bone: They both consist of collagen (18%), non-collagenous proteins (2%), hydroxyapatite (HAp) (70%), and body fluid (10%; all in *weight*/*volume*) [13,21]. DDM scaffolds naturally possess collagen ligands in the form of Arg-Gly-Asp (RGD)-binding sequences, which explains their excellent tissue compatibility, cell attachment capability (which resembles that of osteoblasts), and absorbability for remodeling at the cellular level [12,13,22]. Bessho et al. extracted BMPs from bone and dentin matrix, and concluded that they exhibit the same osteoinductivity in the body [23].

Not many studies have reported on the effects of tooth-derived bone grafted into the M3 defect area. In 2020, De Biase et al. conducted a split-mouth study and found that chair-side-fabricated DDM prevented periodontal dehiscence, but found no significant advantage on periapical radiographs after 6 months [24]. Two clinical split-mouth trials using chair-side tooth bone-conducted in 2020 (15 patients) by Sánchez-Labrador et al. [16] and 2022 (five patients) by Wushou et al. [25] both showed a reduction of probing depth but no bone healing at the distal aspect of M2. Although tooth-derived bone fabricated at the chair side has advantages in that it reduces production times and enables immediate grafting, concerns have been raised about the material quality and cross-infection issues. Since the DDM used in this study was manufactured at an independent institute that follows standardized protocols and ensures consistent quality, graft timing differed depending on whether other teeth were available from a patient to fabricate DDM before their M3 extraction. We found, however, that graft timing did not affect the outcomes of the DDM graft surgery, even though patients had to undergo an additional intervention (Table 2). To improve patient satisfaction, therefore, allogeneic DDM could be expected to alter Auto-DDM and autogenous bone, and there has been a growing body of research that explores its safety and efficacy [26,27,28].

This retrospective study has several limitations. First, the graft and the control group differed in pre-operative defects at M2. Second, the number of patients enrolled in this study was relatively small. Third, we could not confirm whether the follow-up period was long enough to result in the complete remodeling of the cortico-cancellous complex. Fourth, this study did not assess soft tissue outcomes. Within these limitations, the results could have a possible bias due to the small numbers of the control group. Further investigations of M3 extraction are required to evaluate the effect of DDM, including studies using prospective or split-mouth designs with the balanced control group.

## 5. Conclusions

Demineralized dentin matrix significantly improves the distal bone defect of the mandibular second molar by about 76.7% after third molar extraction in middle-aged patients regardless of whether the grafting was performed immediately or 6 weeks after surgery.

## Figures and Tables

**Table 1:** Demographic and clinical information of all included patients.

Patients	Age	Sex	Group	Follow-Up (Months)	Pre-Operative Defect (mm)	Post-Operative Defect (mm)
1	34	Male	Immediate graft	5.5	6.9	2.4
2	34	Male	Immediate graft	3.4	7.2	2.2
3	37	Female	Immediate graft	4.2	6.9	1.2
4	37	Female	Control		5.2	2.8
5	62	Female	Delayed graft	8.6	8.2	2.3
6	47	Male	Delayed graft	3.5	15.7	6.0
7	47	Male	Control		1.7	2.3
8	59	Male	Delayed graft	1.5	13.3	4.4
9	37	Male	Immediate graft	2.6	11.7	1.5
10	31	Male	Immediate graft	7.9	16.5	0.7
11	32	Male	Delayed graft	6.1	7.7	0.0
12	21	Male	Immediate graft	2.6	5.1	0.6
13	21	Male	Control		3.6	2.0
14	41	Male	Delayed graft	4.2	10.8	1.3
15	41	Male	Control		1.4	1.4
16	52	Male	Immediate graft	5.3	10.8	3.1
17	42	Female	Delayed graft	4.2	9.1	1.8
18	43	Female	Delayed graft	4.2	11.5	1.4
19	72	Male	Delayed graft	2.1	8.5	4.6
20	49	Male	Delayed graft	3.2	10.6	4.12

**Table 2:** Clinical outcomes according to the groups.

	Control (*n* = 4)	Graft (*n* = 16)	*p*-Value
Age (year)	39.3 ± 6.3	42.6 ± 13.3	0.645
Follow-up (months)	5.5 ± 2.6	4.6 ± 2.0	0.721
Pre-operative	2.98 ± 1.77	10.02 ± 3.22	0.001 *
Post-operative	2.12 ± 0.59	2.29 ± 1.67	0.959
Improvement (mm)	1.00 ± 1.20	7.74 ± 3.19	0.001 *
Improvement ratio (%)	22.68 ± 26.19	76.70 ± 15.36	0.001 *

* Statistical significance, Mann-Whitney U test.

**Table 3:** The effect of Auto-DDM graft on the distal root of M2 after M3 extraction according to the graft timing and patient sex.

	Graft after Extraction		*p*-Value *	Sex		*p*-Value *
	Immediate (*n* = 7)	Delayed (*n* = 9)		Male (*n* = 12)	Female (*n* = 4)	
Age (year)	34.8 ± 10.1	48.5 ± 12.8	0.059	40.9 ± 13.7	49.0 ± 11.3	0.291
f/u (months)	4.6 ± 2.1	4.5 ± 2.0	0.950	4.2 ± 1.8	5.7 ± 2.5	0.291
Pre-operative defect (mm)	9.70 ± 4.16	10.26 ± 2.59	0.662	10.14 ± 3.58	9.60 ± 1.71	>0.999
Post-operative defect (mm)	1.75 ± 0.99	2.69 ± 2.02	0.573	2.41 ± 1.88	1.83 ± 0.45	0.885
Improvement (mm)	7.95 ± 4.46	7.57 ± 2.14	0.950	7.73 ± 3.50	7.77 ± 2.14	0.885
Improvement ratio (%)	79.50 ± 12.46	74.60 ± 17.76	0.662	75.80 ± 17.03	80.00 ± 7.90	0.769

* Mann-Whitney U test.

## Data Availability

Not applicable.

## Abbreviations

Auto-DDM: autogenous demineralized dentin matrix; DDM, demineralized dentin matrix.
